# Supplementary figures and images for: Palmitic Acid Regulation of Stem Browning in Freshly Harvested Mini-Chinese Cabbage (Brassica pekinensis (Lour.) Rupr.)
Source: Foods. 2023 Mar 5;12(5):1105. doi: 10.3390/foods12051105 (PMC10001398; doi:10.3390/foods12051105)

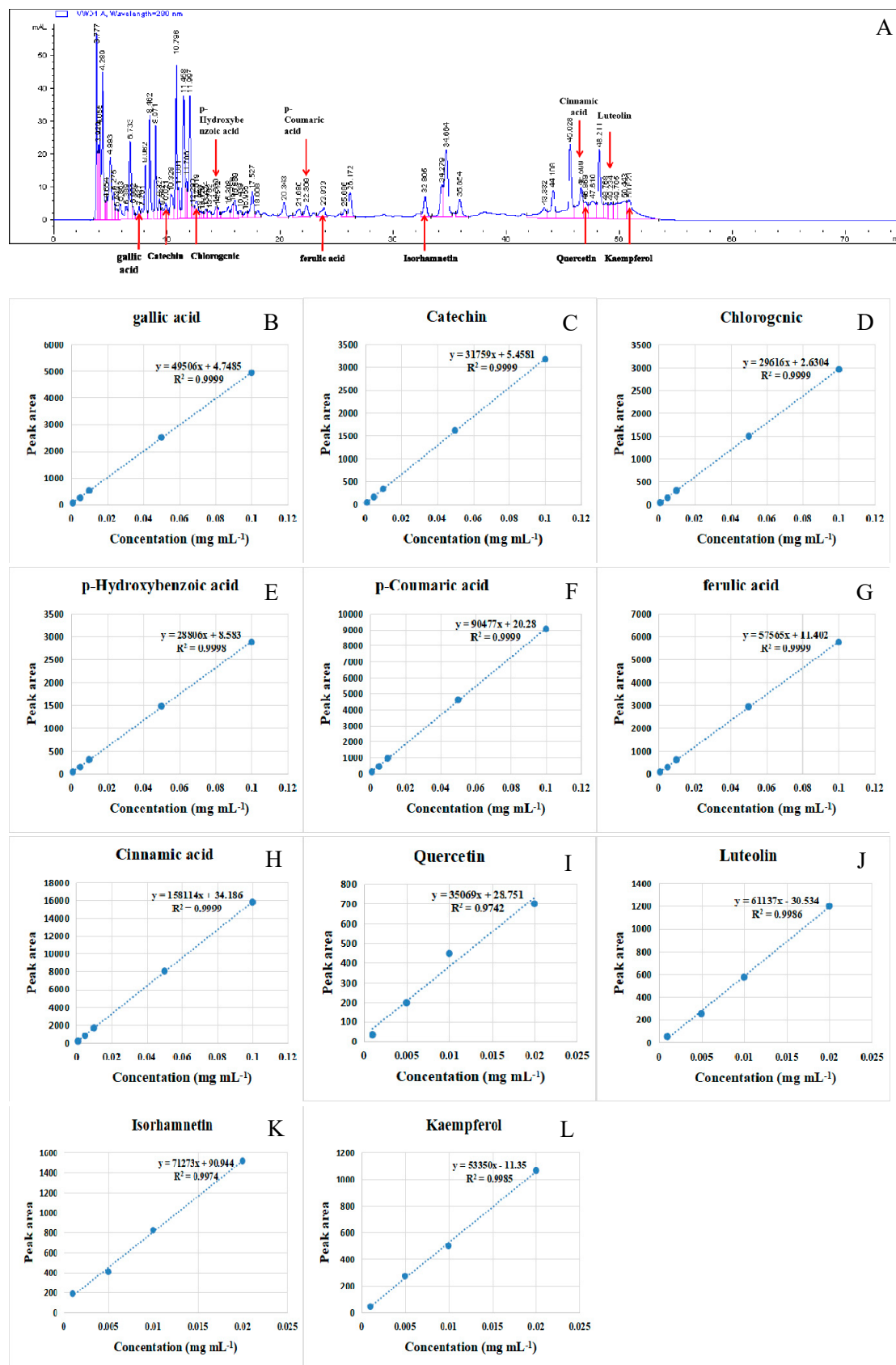

Supplement: Supplementary file 1 [file foods-12-01105-s001.zip › foods-2213180-supplementary.pdf]
